# Application of Crestal Anesthesia for Treatment of Class I Caries in Posterior Mandibular Teeth

**DOI:** 10.5681/joddd.2011.004

**Published:** 2011-03-18

**Authors:** Koroush Taheri Talesh, Shiva Solahaye Kahnamouii

**Affiliations:** ^1^ Professor, Department of Oral and Maxillofacial Surgery, Faculty of Dentistry, Tabriz University of Medical Sciences, Tabriz, Iran; ^2^ Dentist, Private Practice, Tabriz, Iran

**Keywords:** Crestal anesthesia, CT scan, inferior alveolar nerve block

## Abstract

**Background and aims:**

Current infiltration techniques for achieving anesthesia in dental procedures are not applicable in posterior mandibular region because of its dense cortical bone. The aim of this study was to evaluate the efficacy of a specific infiltration anesthesia in posterior mandibular teeth instead of inferior alveolar nerve block for restorative procedures.

**Materials and methods:**

Crestal anesthesia (CA) was assessed both clinically and by computed tomography scan for its efficacy and side effects. A combination of an opaque material (Ultravist) and 2% lidocaine was used to trace the anesthetic solution. The combination was primarily injected in the gingival tissue of rabbit and was followed-up regularly for two weeks to assess any possible injury. After confirming its safety, a combination of these materials was injected to volunteers to assess efficacy and diffusion route. A total of 154 patients (77 female, 77 male) with matched bilateral posterior teeth in mandible were selected randomly and an IANB and CA were performed randomly and separately in different sessions for the contra lateral teeth. The onset of anesthesia, anesthesia duration, pain, blood pressure, pulse rate, and consumed volume of anesthetic solution was recorded for each technique. Data were analyzed using paired t-test.

**Results:**

There were no significant differences in clinical attachment loss, pocket depth, bone level, plaque index, and free gingival margin between the two flaps (p>0.05).

**Conclusion:**

CA could be considered as a reliable and safe primary injection in posterior mandibular teeth for restorative treatments.

## Introduction

Predictable anesthesia is an essential requirement for both the patient and the dentist. The patient’s opinion about the dental treatment is closely related to the local anesthesia experience and the proper use of local anesthesia techniques and pain management are indispensable for successful dental treatment. Although pain control is accomplished successfully in most cases, some anesthesia techniques like mandibular block are accompanied by some drawbacks including difficulty in achieving anesthesia because of anatomic variations;^1^ deep and invasive needle penetration;^2,3^ paresthesia;^1^ muscle trismus;^4^ paralysis;^5^ transportation of oral microbial flora to anatomic spaces;^6^ delayed onset of anesthesia;^7,8^ hematom formation;^9^ high incidence of positive aspiration;^9^ undesired soft and/or hard tissue anesthesia with possible patient-induced injury,^7,10^and difficulty in hemostasis in patients with bleeding disorders.^10,21,24^ The inferior alveolar nerve block (IANB) is the most commonly used injection technique for achieving local anesthesia for mandibular restorative and surgical procedures. However, the IANB does not always result in successful pulpal anesthesia.^21,23^Failure rates of 7 to 75% have been reported in experimental studies.^21-23,25^

Some supplementary anesthetic injection methods have been evolved to circumvent above disadvantages. These include intrapulpal, intraosseous, intraseptal and intraligamentary injections.^1,11,12^ Giffin^13^ introduced crestal anesthesia (CA) as a new variation of intraosseous anesthesia, which he claimed was tested on more than 6000 teeth for different dental procedures ranging from simple restorations to extractions. The technique relies on alveolar crestal perforations formed by canals of Zuckerkandl and Hirschfeld,^14^ which provide gingiva with innervation and circulation. Since, then some have commented on the technique and approved it.^15^ However, to the best of our knowledge, no systematically designed case-controlled study has been proposed to evaluate its benefits and disadvantages. The aim of the present study was to evaluate the time-dependent route and diffused area of anesthetic agent and to compare some clinically-related specifications of IANB and CA to determine their efficiency in posterior mandibular teeth for restorative treatments.

## Materials and Methods

One hundred and fifty four (77 males and 77 females) systemically and mentally healthy individuals, between 26 and 40 years old, were randomly selected from candidates for treatment of class I caries in posterior mandibular teeth during 2008-2010. The patients had bilateral posterior teeth (premolars, or molars) to be restored. The presence of class I caries was confirmed by control periapical radiographs in all patients. Before participation in this study, full medical histories were obtained from all patients and all were physically examined. The subjects were not taking any medication that would alter pain perception. The purpose of the study was explained to the patients and a written informed consent form was obtained from each subject. The study protocol was approved by the Medical Ethics Committee of Tabriz University of Medical Sciences.

### Animal study

The animal study was performed according to NIH guidelines for the care and use of laboratory animals. We used a combination of 1 cc 2% lidocaine with 1:100,000 epinephrine (Darou Pakhsh, Tehran, Iran) and 1 cc of an injectable radiopaque material (Ultravist, Shering AG 13342 Berlin, Germany). As pH of both of these materials was measured almost the same (lidocaine, pH=6.39; Ultravist, pH=6.54), no undesirable reactions were assumed to happen. In order to test any potential hazards from this combination, it was initially injected to the anterior gingiva of rabbit (Oryctolagus cuniculli) in 2 different sites (interdental papilla and attached gingiva). The injection sites were followed up regularly everyday for two weeks to determine any possible desquamation and/or soft/hard tissue necrosis. By the end of the follow-up period, it was judged that the combination has no potential hazard for the gingiva.

### Crestal Anesthesia Technique

A regular dental anesthetic syringe and a standard short 27 gauge needle were used. A topical anesthetic agent (benzocaine) was applied with a cotton-tipped applicator to an interdental gingival papilla adjacent to the tooth. Then the syringe was positioned and the papilla was penetrated, needle bevel and orifice positioned subperiosteally adjacent to bone and crestal nutrient canals. Then, using a significant pressure initially, the anesthetic agent was injected. This procedure should last at least 20 sec, and usually 1.8 mL of the anesthetic agent, equal to a standard anesthetic cartridge, suffices per papilla. One or both (in case of inadequate numbness) of the papillae can be used for the procedure. In this study, we used both papillae to get adequate anesthesia in restorative operations.

### Study design

Using a crossover design, we randomly performed CA and IANB techniques at two separate appointments. CA was used on one side, and a classic direct IANB plus long buccal nerve block was performed in the contra lateral side. Assigned random numbers determined the order of the anesthetic techniques. The two appointments were scheduled at least two weeks apart. Patients were prepared for the operation by skilled hygienists blinded to the study and the CA or IANB techniques were performed by the principal investigator (KTT). In few cases (n=6), in which there was no interdental bone in the contra lateral side because of adjacent tooth/teeth extraction or a periodontal disease was present, an intraseptal anesthesia was administered instead of CA. In this case, we utilized four line angles of tooth to perform intraseptal anesthesia. The patients were followed up for three months and were told to report any probable problem encountered.

Using a written questionnaire, all patients were asked to rate the injection pain based on a scale of 0-5, where 0 represented no pain, 1 mild pain, 2 moderate pain, 3 moderate to sever pain, 4 severe and 5 intolerable pain.

Contra lateral canine was used as the anaesthetized control to ensure that the pulp tester was operating properly and that the subject was responding appropriately during the experiment. At the beginning of each appointment and before any anesthetic administration, trained blinded hygienists tested the experimental tooth and the control canine 3 times using a pulp tester (Gentle-Pulse, Parkell, Farmingdale, NY, USA) to record baseline vitality. After the tooth to be tested was isolated with cotton rolls and dried with gauze, tooth paste was applied to the probe tip, which was then placed midway between the gingival margin and the occlusal edge of the tooth.

The criterion for pulpal anesthesia was an absence of response by the patient to the maximum output (10). The current rate was set at 25 sec. to increase from no output (0) to the maximum output (10). In order to determine the onset of anesthesia, we tested the pulp after the patient’s lip in the injection side was numb in IANB or immediately after second papillary injection in CA. We also tested the contralateral canine to ensure the reliability of testing. We considered anesthesia successful when a subject had two consecutive maximum readings (10). Anesthesia was also not successful if no signs of tissue numbness were observed in IANB within 10 min, or any supplemental injections (such as mental nerve block) was required to gain anesthesia. In order to determine the effective duration of anesthesia, we instructed the patients to raise their hands during or after the operation whenever they sensed any pain, which in this situation they were administered by a second injection of the primary anesthesia technique.

We used a portable digital pulse-oximeter (Onyx 9500, Nonin Medical Inc., Min, USA) to monitor the patients pulse rate during the injection. The instrument has an attachment that patient’s forefinger is placed in it and the heart rate is recorded. An anxiety-reduction protocol was used for all patients to avoid any stress born undesired increase in recording these variables: 10 min before any recordings, the patient was guided to a rest position in a quiet operating room. Then the subjects were asked if they felt ok and whether they felt their heart is beating faster. We tried to reduce the anxiety in our patients by explaining the operation further and answering their questions. Otherwise, they were excluded from pulse rate study. The pulse rate was recorded 5 sec prior the penetration of syringe’s needle and 5 after the injection was terminated. An average of two recordings was used to compare the difference of pulse in two techniques.

To record the changes (increase) of blood pressure, an automatic digital blood pressure monitor (Omron HEM-711AC, Omron Healthcare Inc, Bonnockbum, Il, USA) was utilized. The blood pressure was recorded 5 sec prior to the penetration of syringe’s needle to record the base line blood pressure. Then, we recorded the pressure immediately after the injection was initiated and immediately before its termination. Again an average of two recordings was used to compare the difference of blood pressure in two techniques. In order to compare, the administered volume of the anesthetic solution, anesthetic cartridges were stamped with milliliter marks and the consumed volume was recorded.

Finally, CT scans were obtained from the lower jaws of two volunteers after the CA injection (using the mentioned combination of anesthetic solution and radiopaque agent previously tested on rabbit) to show the solution’s route.

Paired t-test was applied to data to compare each variable between two groups. P value < 0.05 was considered as statistically significant. The SPSS 10 software was used for data analysis.

## Results

Seventy seven female and 77 male patients with an average age of 34.7 years participated in this study. The anesthetic success rates are presented in Table 1. Although not statistically significant, most of unsuccessful CA injections were in the first premolar region.

**Table 1:** Percentage (number) of successful anesthesia achieved by crestal anesthesia (CA) and inferior alveolar nerve block (IANB) techniques

Tooth"	CA	IANB
First premolar	96 (16)	82 (17)
Second premolar	98 (26)	83 (21)
First molar	100 (52)	85 (45)
Second molar	100 (40)	88 (36)
Third molar	100 (19)	93 (15)

There was a statistically significant difference (p<0.001) in the onset of anesthesia between CA (7.00 ± 0.71 sec) and IANB (3.30 ± 0.67 min). A statistically significant difference was also present (p<0.05) between the duration of anesthesia in CA and IANB, which lasted 23.10 ± 2.13 min and 32.10 ± 2.02 min, respectively.

There were no significant differences in heart rate increase between CA (0.58 ± 0.32 beat min^-1^) and IANB (0.97 ± 0.00 beat- min^-1^) (p>0.05). Blood pressure increased 0.00 ± 0.07 mmHg in CA and 0.97 ± 0.00 mmHg in IANB groups, with no statistically significant difference (p>0.05). Only about a fifth of an anesthetic cartridge (0.40 ± 0.07 mL) was consumed in CA. On the other hand, IANB needed about five times more of the anesthetic solution (1.99 ± 0.06mL) for initiating the anesthesia. Most of the pain ratings were in the moderate to severe and severe categories for IANB (3.44 ± 0.22) and moderate to severe category for CA (1.54 ± 0.18) with a statistically significant difference (p<0.001). None of the studied variables showed a statistically significant difference for left and right of the mandible.

**Figure 1:**
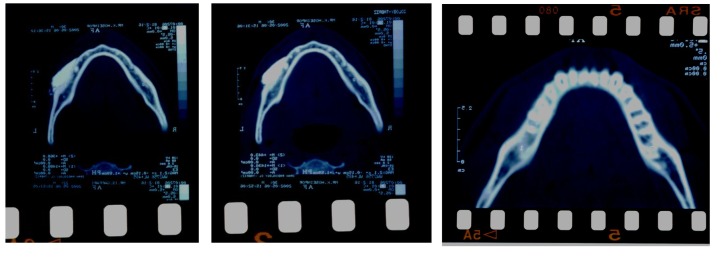
Penetration of radiopaque agent in cancellous bone was seen after the injection in axial CT scan.

The diffusion route of the anesthetic solution can be seen in Figure 1. The opaque area in the injection site is a result of the instant diffusion of the injected media (anesthetic agent plus opaque material).

About half of the patients in the CA group complained from a mild gingival soreness of no more than 1 day. A majority of patients receiving CA were pleased with not having discomfort and incapacitation often experienced with IANB anesthesia. By the end of three month follow up, we found no problem that could be attributed to CA.

## Discussion

Although similar intraseptal injection methods utilizing the alveolar bone nutritional canals are traditionally considered as supplementary injections, they are successfully used by numerous clinicians as a primary route of anesthetic administration. We conducted this study to systematically test the different aspects of CA method.

According to the results, the anesthesia was virtually instantaneous for CA and more lasting in IANB for the restorative operations. It was interesting that in some cases with CA patients with a prior experience of IANB did not realize the completion of the injection. An ooze was sensed during a successful CA injection that clinically guarantied the fast onset and a single administration of the anesthetic agent.

CA can be placed in the midway of conventional intraosseous anesthesia and infiltration techniques. Karna^15^ called the technique intraosseous pressure anesthesia that although correctly describes its forceful nature but might be misleading because it really does not penetrate the bone.

The benefits of conventional intraosseous injections (IOI) are clearly known. With the advances in this area and introduction of new instruments and techniques, patients and dentists benefit from profound anesthesia without unnecessary lip and tongue anesthesia. Despite this, IOI has not been as popular as the infiltration and block techniques, and the reason has been associated with the reluctance of dentists to drill a healthy soft and hard tissue and in some cases the difficulty of inserting a needle precisely into the drilled hole.^16^

Considering the maximum reading of the pulp tester (here 10) for a successful anesthesia was due to the fact demonstrated by previous studies that the criterion was necessary for performing a painless restorative treatment.^17,18^ More unsuccessful injections in the premolar region might be due to dense cortical bone of mental foramen that acts like a dam and reduces the diffusion rate of anesthetic solution. Reduced diameter and fewer nutrient canals compared to posterior region might also play a role. Reported primary intraligamentary anesthesia success rates of 74-92% were observed in CA (99%),^7,10,19^ that might be due to subperiosteal nature of CA. Giffin^13^ believes that in this method the injectate is captured over a broader bone area, allowing access to more alveolar crest nutrient canal foramina, despite the apparently greater relative number of nutrient canal foramina in tooth socket cribriform plate. It seems that the high success rate of CA is due to fast (or even immediate) diffusion of anesthetic agent through the very porous region of the dental socket. This fact is also confirmed by other series of CT scans (same exposure angle, near successive times).

Longer duration of anesthesia in IANB compared to CA was an expected finding. CA produced duration of anesthesia similar to those of reported intraligamentary injection.^20^ This was expected because both methods rely on perfusion of medullary bone. The most successful IANB injections were in the third molar region. This could be because of supplemental long buccal injection and shorter distance of the tooth to the injection site.

We observed a case of buccal tissue anesthesia in the block group due to long buccal nerve anesthesia. CA required considerably less than one full cartridge of anesthetic agent. This reduced amount of anesthetic significantly reduces the bolus of epinephrine when compared to conventional block techniques such as IANB. Another advantage of CA is its 0% of positive aspiration. The above facts might explain the reason for the statistically lesser readings of the blood pressure and the pulse rate. Indeed, many patients are less acquainted with the CA at least as a primary technique but have experienced the IANB many times before. A single bad experience with the IANB might be enough for an increased anxiety and the resultant systemic effects. Longer penetration time (before the deposition of the anesthetic agent) and possible changes in the needle direction by the clinician to meet the required clinical landmarks is another reason for the increased anxiety.

As with intraosseous types of injections, the CA allows bilateral treatment of mandibular areas without complete mandibular numbness and lack of tongue control.

CA injections penetrate the uncomplicated tissue structures aseptically that probably accounts for mild post injection discomfort (gingival soreness).^13^ The presence of anatomical anomalies such as tori at the proposed site of injection would preclude the dentist from using the CA effectively. This contraindication might include the situations where a medullary bone density variation (for example condensing osteitis) is present. The mentioned factors necessitate accurate clinical and radiographic surveys prior to the selection of the injection technique.

All patients were pleased that the CA did not interfere with tongue and lip sensation compared with IANB. Patient’s ability to return to their regular daily routines immediately postoperatively with oral tissues that feel normal can increase the perceived volume of this technique for the dentists and the patients.

## Conclusion

Crestal Anesthesia is an efficient, fast, and reliable technique in posterior mandibular dental restorative procedures and could be considered as a reliable and safe primary injection in posterior mandibular teeth for restorative treatments.
